# Highlight: More Than Meets the Eye—Nonvisual Opsins Are Crucial for Light-Sensing in Vertebrates

**DOI:** 10.1093/gbe/evaf075

**Published:** 2025-05-14

**Authors:** Pedro Andrade

**Fig. 1.**
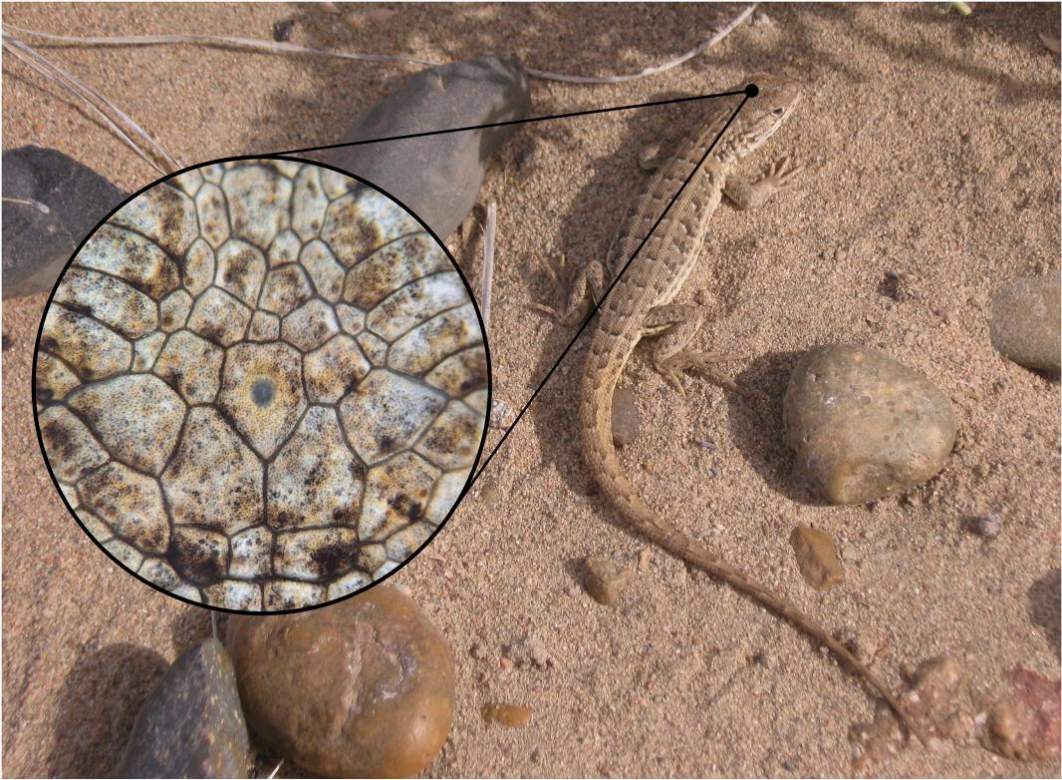
A Patagonian lizard (*Liolaemus darwinii*) in its wild habitat near Puerto Madryn, in the Argentine Patagonia (photo by Ángeles Amette-Estrada). The inset is of the dorsal side of the head of a *L. darwinii* specimen, showing the parietal scale with the third eye underneath (photo by Ricardo Romero and Flávio S. J. de Souza).

The evolution of eyes, through the co-option of photosensitive proteins known as opsins, represents a major innovation that expanded the ecological and communication capacities of animals (Hagen et al. 2023). Five distinct “visual opsins” underlie light-sensing in the retinas of vertebrate eyes. However, animal genomes contain many “nonvisual opsin” genes that share a close evolutionary history with the five visual opsins. These are expressed in the so-called pineal complex, a simple light-sensing organ located on the top of the head, which in some vertebrate lineages can even resemble a “third eye” (known as the parietal eye). Among “reptiles” (i.e. snakes, lizards, and crocodiles, but excluding birds), the parietal eye is only found in the New Zealand tuatara (*Sphenodon punctatus*) or in some lizards such as the South American iguanids of the genus *Liolaemus* (Fig. 1).

At the start of their project, Ricardo Romero and Flávio S. J. de Souza—researchers at the Universidad de Buenos Aires, Argentina—were immediately surprised by what they found when studying the visual anatomy of *Liolaemus*. They shared “even though we knew from the scientific literature what this structure should look like, we were astonished by how much it resembles a tiny lateral eye, complete with a lens, retina, parietal nerve […] despite being in the ‘wrong’ place on the head.”

Non-visual opsins, such as those in parietal eyes, can sense light. They are involved, for example, in regulating day/night physiological cycles. So, could their evolution help researchers understand how light-sensing has evolved in vertebrates? Two new studies published in *Genome Biology and Evolution*, by Romero and de Souza (2025) and Gyoja et al. (2025), traced the evolutionary history of nonvisual opsins, leveraging the growing collection of vertebrate genomes. Romero and de Souza focused on a detailed description of the evolution of these genes in lepidosaurs, the group that includes lizards, snakes, and the tuatara, while Gyoja and colleagues queried 451 vertebrate genomes for the presence of non-visual opsin genes.

Previous studies suggested the existence of four nonvisual opsins that were particularly similar to the five visual opsins. Romero and de Souza (2025) started their study by locating these four opsins and found them in many of the 63 reptile genomes they surveyed. The first big surprise in their study was their detection of a novel non-visual opsin gene—named lepidopsin—that appeared in most of the reptile genomes. The evolution of lepidopsin, as revealed by genomes of reptiles and other vertebrates, was not exactly straightforward. While it is absent in most terrestrial vertebrates, fragments of its coding sequence are found in several ray-finned fish and the coelacanth, and, of course, in many lizards. This suggests that this gene originated over 430 million years ago, during the Silurian period and before the split of ray and lobe-finned fish (Brazeau and Friedman 2015), and then lost its function and degenerated multiple times, generating its “patchy phylogenetic distribution.” These findings match those of Gyoja et al. (2025), who found functional and nonfunctional copies of this gene (named QB opsin by these authors) across bony fish and tetrapod genomes but none in jawless fish or sharks. Did the first bony fish have something akin to a parietal eye? Consistent with this hypothesis, multiple fossil species of Silurian and Devonian fish and early tetrapods had a bony opening in the skull roof where the parietal eye was likely located.

The fate of lepidopsin and the other non-visual opsins seems tightly linked to the evolution of light-sensing, as revealed by their trajectories throughout lepidosaur evolution. While looking at the genomic distribution of the now five non-visual opsins, Romero and de Souza (2025) noticed their genomic presence was associated with the anatomical presence of the light-sensing pineal complex. While not all five “pineal” opsins were present across all species of reptiles, this repeated overlap and previously reported expression patterns make it highly suggestive that they have served—and continue to serve—a function in light-sensing throughout vertebrate evolution. Illustrative of this, the tuatara itself conserves all five non-visual opsins in its genome. On the flip side, loss of these genes was most common in either nocturnal species or those with a burrowing lifestyle, for which losing light sensitivity is less of a constraint. According to de Souza, “when a lizard group lacks the parietal eye, it will also lack the nonvisual opsins parapinopsin, lepidopsin, and parietopsin. This is true for geckos, whiptails, worm lizards, beaded lizards, and snakes, all of which lost the parietal eye independently, in a striking case of convergent, regressive evolution.”

Romero and de Souza have already set their sights on understanding the expression patterns of lepidopsin, as de Souza explains: “is it really expressed in photoreceptors of the parietal eye? Does it co-localize with the other opsins in the same or different photoreceptor cells?”. One challenge remains the relative paucity of genomic resources of reptiles, as de Souza concludes: “Squamates (lizards and snakes) are quite varied in morphology, habitats, behavior, sensory capabilities, and mode of reproduction; genome resources should encompass more of lizard and snake diversity.”

*Want to learn more?* Check out these other articles on opsin genes and vertebrate vision recently published in *Genome Biology and Evolution*:

- Powell A, Heckenhauer J, Pauls SU, Ríos-Touma B, Kuranishi RB, Holzenthal RW, Razuri-Gonzales E, Bybee S, Frandsen PB. Evolution of opsin genes in caddisflies (Insecta: Trichoptera). Genome Biol Evol. 2024:16(9): evae185. https://doi.org/10.1093/gbe/evae185.
- Torres-Dowdall J, Karagic N, Prabhukumar F, Meyer A. Differential regulation of opsin gene expression in response to internal and external stimuli. Genome Biol Evol. 2024:16(7): evae125. https://doi.org/10.1093/gbe/evae125.
- Rossetto IH, Sanders KL, Simões BF, Van Cao N, Ludington AJ. Functional duplication of the short-wavelength-sensitive opsin in sea snakes: evidence for reexpanded color sensitivity following ancestral regression. Genome Biol Evol, 2023:15(7): evad107. https://doi.org/10.1093/gbe/evad107.
